# Invariant properties of upper limb movement trajectory control

**DOI:** 10.1088/1741-2552/ae8bd8

**Published:** 2026-08-05

**Authors:** Shriniwas Patwardhan, Jonathon S Schofield, Wilsaan M Joiner, Siddhartha Sikdar

**Affiliations:** 1 Department of Bioengineering, George Mason University, Fairfax, VA 22030, United States of America; 2 Mechanical and Aerospace Engineering Department, University of California, Davis, CA 95616, United States of America; 3 Department of Neurobiology, Physiology and Behavior, University of California, Davis, CA 95616, United States of America

**Keywords:** motor planning, minimum-jerk trajectory, myoelectric control, visual feedback, effector independence, motor invariance, human–machine interface

## Abstract

*Objective.* Human movements often follow smooth trajectories predicted by the minimum-jerk trajectory (MJT) model, suggesting that the motor system optimizes motion smoothness during planning. While MJT-like behavior has been extensively studied in reaching movements, it remains unclear whether these trajectory properties are preserved across different control modalities, visual feedback conditions, and limb status. *Approach.* We examined the effects of control modality (reaching versus muscle-based control), visual feedback (present versus absent), and limb status (intact limb versus limb difference) on movement trajectory generation. Thirteen participants, including three individuals with upper-limb difference, performed virtual cursor movements to varying target distances. Peak velocity, movement time, position error, and adherence to MJT-like trajectory structure were analyzed. *Main results.* Across all experimental conditions, movements exhibited bell-shaped velocity profiles and amplitude-dependent scaling of peak velocity and movement time. These features persisted in the absence of visual feedback and in participants with limb difference. Position error increased when visual feedback was removed, but the overall smoothness and structure of the trajectories remained consistent with MJT predictions. *Significance.* These findings suggest that MJT-like movement structure is robust across variations in control modality, visual feedback availability, and limb status. The results support the idea that smooth movement planning reflects a generalized property of motor control and provide insight for the development of biologically inspired assistive and human–machine interface systems.

## 1. Introduction

Human movement demonstrates some key typical features, one of which is the adherence to smooth, coordinated trajectories that are thought to reflect internal optimization processes within the motor system. A foundational theory in this domain, proposed by Hogan and colleagues [1], suggests that the central nervous system minimizes the third derivative of position (jerk) when planning point-to-point movements. This optimization leads to the minimum-jerk trajectory (MJT), which is obtained by minimizing the integral of the squared jerk over the duration of movement:

\begin{align*} \mathrm{MJT} = \int_{t_\mathrm{s}}^{t_\mathrm{f}} ||\dddot{x}\left(t\right)||^2\, \mathrm{d}t.\end{align*}

Solving this optimization yields analytically defined position and velocity profiles [2]:

\begin{align*} x\left(t\right) = x_0 + \left(x_{f} - x_0\right)\left(10\tau^3 - 15\tau^4 + 6\tau^5\right),\end{align*}

\begin{align*} v\left(t\right) = \frac{1}{d}\left(x_{f} - x_0\right)\left(30\tau^2 - 60\tau^3 + 30\tau^4\right),\end{align*}

where $x(t)$ and $v(t)$ denote position and velocity at time $t$, $t_\mathrm{s}$ and $t_\mathrm{f}$ denote start and finish times, $d = t_\mathrm{f} - t_\mathrm{s}$ is the movement duration, $x_0$ and $x_{f}$ represent the start and end positions, and $\tau = (t - t_\mathrm{s})/d$ is normalized time. These equations define the smooth sigmoidal position and bell-shaped velocity profiles characteristic of natural movement.

Subsequent studies have demonstrated that MJT-like behavior is not limited to arm reaching but is also observed across a range of behaviors, including vertical arm movements, drawing, catching, head movements, chewing, and saccadic eye movements [3–8]. These findings suggest that the nervous system may apply a consistent planning strategy that generalizes across joints, effectors, and tasks. In addition, MJT-like behavior has been reported during both simple and complex multi-joint upper-limb movements [9, 10]. The presence of bell shaped velocity profiles and MJT-like trajectories mainly during point-to-point reaching movements has been extensively documented, including in the seminal works of Flash and Hogan [1, 10], which demonstrated that unconstrained reaching movements retain smooth trajectory structure even when direct visual information about the arm is reduced. These studies established that movement trajectories exhibit approximately straight hand paths and bell shaped tangential velocity profiles consistent with minimum jerk predictions. Subsequent studies have similarly demonstrated that these invariant movement features are preserved across a variety of motor tasks and sensorimotor conditions. Most studies of MJT-like trajectories focus on limb kinematics, leaving open the question of whether these smooth movement patterns persist when movement is produced without a limb, using alternative biosignals as the control signal.

In our recent work [11], we addressed this gap by examining whether MJT-like behavior is preserved when the control signal is derived directly from muscle activity rather than from overt limb motion. Using sonomyography [12–17], a technique that measures real-time muscle deformation via ultrasound imaging, we developed a proportional control interface in which participants generated one-dimensional cursor movements by modulating deep and superficial flexor muscles of the forearm during wrist flexion and extension movements. This framework allowed us to characterize movement trajectories within a control domain that precedes overt kinematic execution and is directly associated with muscle activation. While sonomyography served as the measurement modality in our previous work, our objective was not to evaluate the utility of sonomyography per se, but rather to determine whether control derived from muscle-based activity adheres to the same fundamental principles of motor coordination observed in conventional reaching.

We found [11] that the trajectories produced through muscle-based control exhibited hallmark features of the MJT model, reflecting reaching behavior. Cursor paths displayed smooth bell-shaped velocity profiles with a single velocity peak near the midpoint of movement, and both peak velocity and time-to-target scaled linearly with movement amplitude, mirroring classical findings from point-to-point reaching. Furthermore, position traces showed minimal corrective submovements, indicating that participants were able to generate smooth and coordinated muscle activations even in the absence of explicit joint motion. These properties were consistent across participants and target distances, suggesting that smoothness-based planning may arise from mechanisms that are deeper than just the biomechanics of limb segments. In addition, our analyses showed that muscle deformation trajectories closely matched theoretical MJT predictions when quantified using root-mean-square error and temporal alignment metrics. This agreement was comparable to that observed in traditional reaching tasks, despite the fundamentally different control domain (muscle-based control vs kinematic control). Together, these results provided the first evidence that smoothness optimization may be expressed at the level of muscle activation itself, offering a strong rationale for using muscle-based techniques such as sonomyography to investigate motor control policies.

While that work demonstrated that smooth, MJT-like trajectories can emerge from muscle-based control, some critical questions remained unexplored regarding the factors that shape and sustain such behavior.

It remained unclear how sensory feedback (visual and proprioception) and the presence of an intact limb shaped the emergence of smooth trajectories during muscle-based control. Many classical studies of reaching and movement kinematics have included continuous visual feedback of the end-effector, which may have contributed to online corrections and refinement of behavior. It remained unclear whether smoothness persists in the absence of visual information, or whether MJT-like trajectories break down when visual guidance is removed.

A second important consideration is the presence or absence of proprioceptive and kinesthetic feedback from the moving limb. Although movement smoothness can be disrupted at the kinematic level in certain movement disorders, muscle-level MJT-like trajectories have been shown to remain consistent in individuals with spinal cord injury [18]. However, these individuals still possess an intact limb, even if sensorimotor processing is impaired. It remains unclear how these dynamics manifest in individuals with limb difference. Do movements executed with a prosthetic limb follow the same MJT-like profiles? And do these individuals also exhibit muscle-level MJT-like patterns similar to those observed in people with intact limbs or spinal cord injury? For individuals with limb difference, muscle-based control remains viable because residual musculature can still be voluntarily activated [12, 19–21]. Moreover, they can generate kinematic movements using prosthetic devices that enable interaction with the environment, albeit through artificial effectors. These movements, however, typically lack natural afferent sensory feedback from the missing joints, tendons, and cutaneous receptors. As a result, haptic, tactile, and kinesthetic information that normally informs and refines internal movement representations is absent. Whether such individuals can nonetheless produce reaching trajectories or muscle-level activation patterns that resemble those of individuals with intact limbs is not well understood. This question is central to understanding the relative contributions of sensory feedback to motor coordination and trajectory formation. Prior work has shown that individuals with limb difference can use muscle-level signals to control virtual cursors without extensive training [21]. These findings suggest that the absence of limb-based sensory input does not preclude the use of internal models to guide movement and motivate further investigation into how feedforward and feedback processes together shape motor performance in this population.

A third related question is whether the modality of control (controlling a cursor by reaching with the limb versus controlling it via muscle-based signals) affects the emergence of smooth, coordinated behavior. While muscle-based control preserves some aspects of volitional activation, it is divorced from joint movement and lacks many of the mechanical constraints associated with limb dynamics, especially in individuals with limb difference. This raises the possibility that smooth trajectories observed in muscle level tasks may reflect learned output mappings, or may be influenced by feedback-based corrections rather than intrinsic planning. Although this question was somewhat examined in our prior work [11], here we explore the effects of the interactions between the control modality and the presence of multi-level multi-modal feedback.

Collectively, the present study addresses a fundamental question in motor control: do smooth, bell-shaped movement trajectories emerge from internal feedforward mechanisms, or are they primarily shaped by the interaction between motor intent and sensory feedback, particularly vision? They also highlight the importance of understanding how the MJT framework extends across control modes, populations, sensory feedback conditions, and the presence or absence of a physical effector. Determining whether MJT-like trajectories persist when actions are carried out with or without a limb, and under different sensory feedback conditions, should guide the development of motor interfaces and assistive technologies.

To summarize, the present study is novel and builds upon prior work in three important ways. First, we investigate MJT-like behavior in individuals with limb difference, including both prosthesis mediated reaching and residual muscle based control, allowing us to examine whether canonical trajectory structure is preserved under altered limb configurations. Second, we examine movement generation under transformed sensorimotor mappings in which cursor motion is not directly equivalent to hand motion in external space, unlike conventional reaching paradigms. Third, we investigate MJT-like behavior during muscle based sonomyographic control, allowing trajectory formation to be studied at the level of muscle deformation rather than overt limb kinematics. Together, these extensions allow us to test the generalizability of smoothness based motor planning across altered embodiment conditions, transformed control spaces, and alternative control modalities.

In this work, to address the questions raised above, we designed an experiment aimed at systematically dissociating the roles of sensory feedback, limb presence, and control modality in shaping movement trajectories. Participants performed a virtual target acquisition task where we varied control modality (muscle-based control vs reaching), and presence visual feedback (ON vs OFF), and analyzed the control trajectories exhibited by the participants. We hypothesized that adherence to MJT characteristics such as bell-shaped velocity profiles and amplitude-scaled peak velocities would be preserved across all conditions. Specifically, we predicted that participants would produce MJT-like profiles regardless of whether control was exerted through joint movement or muscle activation, whether visual feedback was present or absent, and whether the limb was intact or missing. This hypothesis reflects the idea that MJT behavior arises from a robust, generalized internal motor planning strategy, rather than from the availability of visual or proprioceptive feedback.

## 2. Materials and methods

### 2.1. Participants

Ten individuals with intact limbs and three individuals with upper-limb difference were recruited for this study. All experiments described in this work were approved by the George Mason University Institutional Review Board and performed in accordance with relevant guidelines, regulations, the principles embodied in the Declaration of Helsinki, and local statutory requirements. All participants provided written informed consent prior to participating in the study and were compensated for their participation.

Among the three participants with limb difference, one participant had a congenital limb difference and was not a prosthesis user. A second participant had approximately 29 years of experience using a dual site myoelectric prosthesis. The third participant had approximately 9 years of experience using multiple prosthetic control systems, including dual site myoelectric control, myoelectric pattern recognition, and body powered prostheses.

### 2.2. Experimental implementation

#### 2.2.1. Conditions

**Figure 1.**
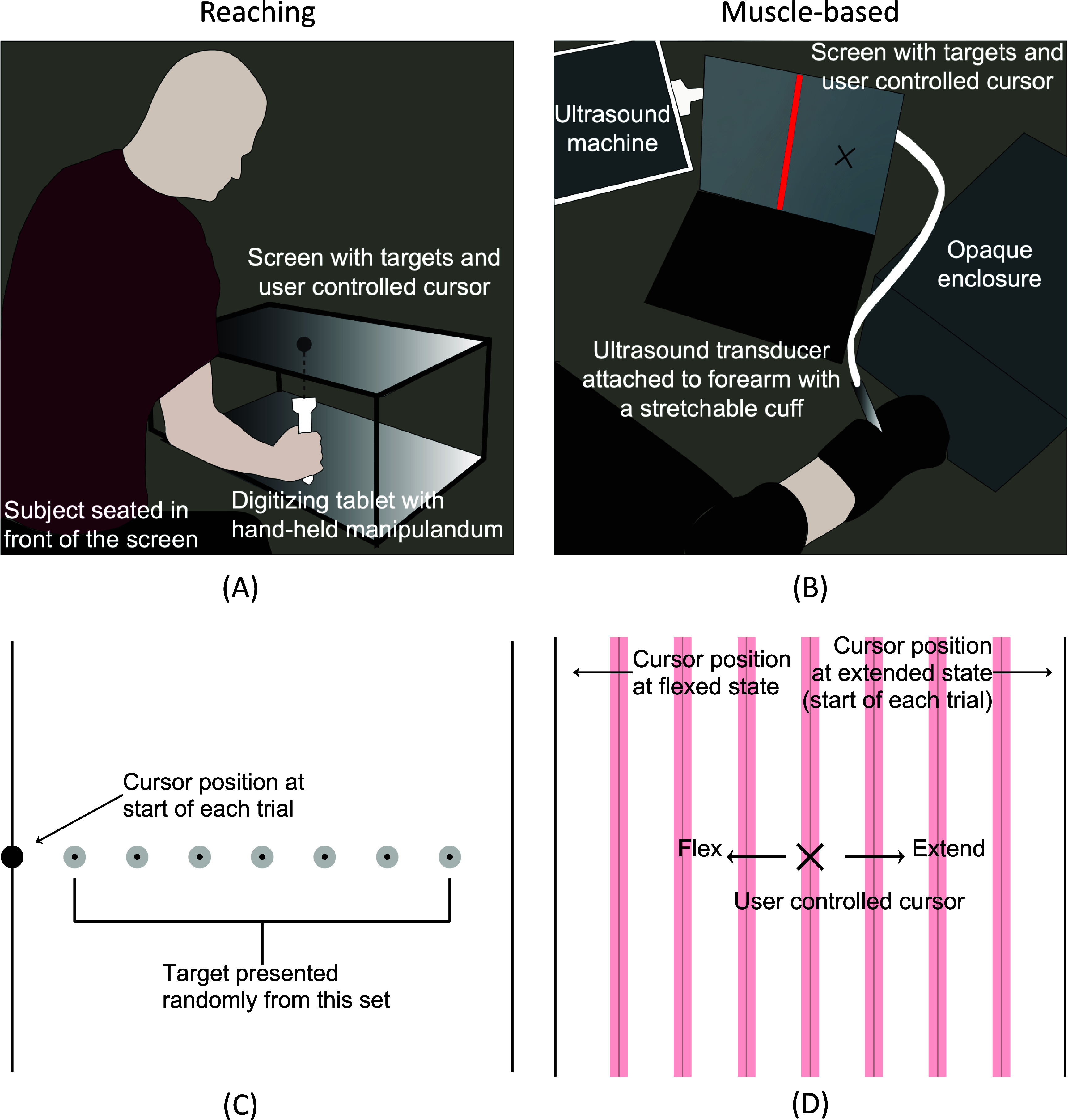
(A) Setup for reaching. Participants were seated in front of a digitizing tablet to capture the movement trajectories as they moved a manipulandum. A LCD monitor was placed above the tablet that showed the target location and the participants’ manipulandum position. The participants were asked to achieve the target positions displayed on the screen.(B) Setup for muscle control. Participants were instrumented with an ultrasound transducer that recorded cross-sectional ultrasound images of their forearm, which resulted in a proportional signal correlating to their extent of flexion. The position of the participant-controlled cursor on the screen in front of them was driven by the proportional signal derived from sonomyography. Participants were asked to achieve the target positions displayed on the screen.(C) Diagram showing the target acquisition task performed during reaching. The dark circle on the left shows the starting position, while all the possible target locations are shown as faint circles on the screen.(D) Diagram showing the target acquisition task performed during muscle control. All possible targets are given by faint red lines. The ‘$X$’ indicates the participant-controlled cursor. A fully extended wrist state would correspond with the cursor being at the right-most edge of the screen and a fully flexed state of the wrist would correspond with the cursor being in the left-most edge of the screen (for a right handed participant). Reproduced from [11]. CC BY-NC-ND 4.0.

The experimental design incorporated three key binary factors: (1) control modality, comparing control of a cursor via traditional arm based reaching to sonomyographic forearm muscle activation; (2) visual feedback, comparing trials in which participants could view the real time position of the cursor to trials in which this information was withheld; and (3) limb status, comparing participants with intact upper limbs to individuals with acquired upper limb difference. This 2 $\times$2 $\times$2 factorial design produced eight experimental conditions labeled A through H (table 1, figure 1). By varying these three factors independently, we sought to examine whether MJT-like trajectory formation is preserved across altered embodiment conditions, transformed control mappings, and different sources of sensory feedback. Specifically, this design allowed us to compare conventional reaching movements with muscle based control generated through transformed sonomyographic signals, while also evaluating whether these trajectory properties are preserved in individuals with limb difference and under reduced visual feedback conditions.

**Table 1.** Eight experimental conditions defined by combinations of visual feedback, control modality, and participant type. This $2 \times 2 \times 2$ design explores how different feedback and control pathways affect trajectory performance in individuals with and without limb difference.

Condition	Visual feedback	Control modality	Participant type
A	Yes	Reaching	Individuals with intact limb
B	Yes	Reaching	Individuals with limb difference
C	Yes	Muscle control	Individuals with intact limb
D	Yes	Muscle control	Individuals with limb difference

E	No	Reaching	Individuals with intact limb
F	No	Reaching	Individuals with limb difference
G	No	Muscle control	Individuals with intact limb
H	No	Muscle control	Individuals with limb difference

#### 2.2.2. Data collection setup

The experimental apparatus and data acquisition procedures were identical to those used in our previous study [11], and reproduced here (figure 1). In summary, for reaching, participants used a manipulandum-based interface in which hand movement on a digitizing tablet controlled an on-screen cursor, with the visual display occluding the arm. For muscle-based control, participants used a sonomyography-based interface in which real-time ultrasound imaging of forearm muscle deformations was used to drive proportional cursor movement. In both modalities, movement trajectories were analyzed post hoc. The full hardware specifications, signal processing pipeline, and calibration procedures, which follow the same methodology described in our prior publication [11], are detailed in the sections below. The only aspects that differed from the prior study were the presence of visual feedback of the cursor and whether participants had an intact limb.

**Reaching control.** Participants were seated upright at a waist-high table in front of a horizontal 27-inch LCD monitor. Chair height was adjusted to ensure comfortable viewing and reach. The setup consisted of a horizontal display, a digitizing tablet positioned 10 cm below the screen (Intuos4, Wacom), and a PC running the experimental paradigm in PsychoPy. The screen displayed start and target positions; the tablet recorded hand position at 60 Hz. Participants grasped a cylindrical handle, which housed the stylus, and moved it across the tablet. The horizontal screen blocked direct vision of the limb and the tablet. This configuration is referred to as the manipulandum setup. Participants began each trial by moving the handle to a start circle (diameter 5 cm) at the left edge of the screen and holding for three seconds. A target then appeared to the right, and the participant moved to the target circle (diameter 5 cm) and held that position for three seconds before returning to the start location. Seven targets were tested per block, and each block was repeated five times. Participants with intact limbs used their right hand. Participants with limb difference used their prosthesis to perform the same reaching task. Movement trajectories were sampled at 60 Hz and stored for offline analysis. Visual feedback manipulation was applied by showing or hiding the cursor during movement execution. When feedback was absent, participants moved the manipulandum without seeing their cursor position until the end of the movement, but were still able to observe the target.

Participants with limb difference performed the reaching task using their prosthetic device to interact with the manipulandum. The prosthesis was used to securely hold the handle, and cursor movement was generated using proximal limb movements resulting in distal movements of the manipulandum. Depending on individual differences and prosthesis configuration, participants may also have engaged elbow and trunk movements to achieve the required cursor displacement. Movement strategies were not explicitly constrained, and participants were instructed to perform the task in a natural and comfortable manner. Although participants with limb difference did not have access to distal tactile feedback from the prosthetic hand, they retained proximal proprioceptive feedback from the shoulder and residual limb. In addition, incidental feedback from the socket interface, such as pressure and contact forces, may have contributed to their perception of limb position during task execution.

**Muscle-based control.** For muscle-based control, participants sat upright with the dominant forearm placed comfortably on a padded support. A clinical ultrasound system (Terason uSmart 3200 T) with a 16HL7 linear transducer was positioned on the volar forearm to image deep and superficial flexor muscles. The probe was secured with a custom-designed holder and stretchable cuff. Ultrasound images were streamed to a PC (Dell XPS 15 9560) using a USB-based video grabber. Real-time processing in MATLAB [11–13] computed a proportional control signal corresponding to the participant’s wrist flexion and extension. This signal controlled a horizontal cursor displayed on a 27-inch monitor. The cursor moved left or right in proportion to muscle activation. Left- and right-handed participants controlled the cursor in mirrored directions according to their dominant limb. Before performing the main task, participants completed a guided training sequence consisting of timed wrist flexion and extension movements. Ultrasound images collected at full flexion and full extension were averaged into reference states, which defined the two extremes of the cursor’s workspace. All intermediate muscle configurations mapped proportionally to intermediate cursor positions. During each trial, one of seven evenly spaced target positions appeared on the screen. Participants were instructed to move the cursor from a rest position to the target location as accurately as possible within a ten-second window. Each block consisted of seven targets presented once, and participants performed six blocks in total (the first block served as practice). As in the reaching modality, cursor visibility during the movement depended on the visual feedback condition. In feedback-absent trials, the cursor was hidden while the target was visible. During the muscle-based control task, participants generated cursor movement through voluntary wrist flexion and extension, rather than strictly isometric contractions. The control signal was derived from changes in muscle deformation captured via ultrasound imaging. As a result, participants had access to proprioceptive feedback associated with joint motion and muscle activation during task performance.

#### 2.2.3. Computation of movement parameters

Movement trajectories were recorded along with timestamps and imported into MATLAB for analysis. All recorded trials were included.

**Movement distance.** Seven target positions were located at 12.5%, 25%, 37.5%, 50%, 62.5%, 75%, 87.5% of screen width. In both modalities, the cursor workspace was normalized relative to the physical screen width, and movement distance was defined as the displacement of the cursor from the start position (0%).

**Trial start and end points.** Movement onset was defined as the first sample with positive movement velocity. Trials ended when the cursor stabilized for one second at a position between $\pm$10% of the expected position.

**Time to target.** Time to target was the duration from movement onset to the end point. Average time-to-target values per movement distance were used to compute the minimum jerk trajectory.

**Minimum jerk trajectory.** MJT was computed according to established formulations (equations (1)–(3)), representing the trajectory that minimizes squared jerk over the movement duration.

**Position error.** Position error for each trial was defined as the difference between the desired target location and the achieved final resting position (average of the last 5 seconds of the trial).

**Target completion rate.** As described above, a trial was considered successfully completed if the cursor remained within $\pm$10% of the target position continuously for at least 1 second. The target completion rate was defined as the percentage of trials meeting this criterion within each condition.

#### 2.2.4. Statistical analysis

All recorded trials were included in the analysis.

**Scheirer–Ray–Hare (SRH) test.** To analyze performance across experimental conditions, we conducted nonparametric SRH tests on four response variables: peak velocity, time to target, position error, and time to peak velocity. SRH is a rank-based alternative to multifactorial ANOVA and is robust to violations of normality and unequal sample sizes, making it appropriate for our factorial design, which included a small sample of individuals with limb difference and unbalanced group sizes. We selected SRH over linear mixed-effects models for two main reasons: (1) the limited number of participants with limb difference resulted in unbalanced group cells, potentially compromising the validity of mixed-effects estimation; and (2) non-normality of residuals across several response variables, violating the assumptions of parametric inference. SRH tests were used to assess the effects of control modality, visual feedback, presence of limb, and movement distance on MJT behavior.

All main effects and interactions were included. A significance threshold of $\alpha$ = 0.05 was applied for all comparisons. Given that SRH operates on rank-transformed data and can be less robust for higher-order interactions, all three- and four-way interactions were interpreted cautiously and used primarily to guide exploratory follow-up analyses rather than for definitive inference. All statistical analyses were performed using individual trial data rather than subject-level averages. Each participant completed 10 trials for every combination of movement distance and experimental condition (A–H), yielding a large number of observations per condition. Rank-transformed trial-level data pooled across participants were analyzed using the SRH test. Although this approach improves statistical power, particularly for repeated-measures data, findings involving the limb-difference group ($n$ = 3) should be interpreted cautiously due to the limited sample size.

**Linear regression for distance scaling.** To assess how movement distance influenced behavior, we performed eight linear regressions (one per condition) for each of the following outcome measures: peak velocity and time to target. Each regression modeled the outcome variable as a function of target distance, yielding a slope estimate that characterized the scaling relationship for each condition (A–H). This allowed us to directly compare how movement amplitude-dependent modulation of speed and timing varied as a function of control modality, feedback availability, and limb status.

## 3. Results

We evaluated participant performance (table 3) across eight experimental conditions (A–H, table 1), which systematically varied control modality (reaching vs muscle control), visual feedback (present vs absent), and participant type (intact limb vs limb difference). In all conditions, participants moved a virtual cursor to one of seven target positions (table 2).

**Table 2.** Factors included in the Scheirer–Ray–Hare (SRH) analysis. Each factor was tested independently and in interaction to assess its influence on movement trajectory properties.

Factor	Levels
Control modality	2 levels: Reaching via hand-held manipulandum, Muscle control via sonomyography
Visual feedback	2 levels: Present, Absent
Participant type	2 levels: Intact limb, Limb difference
Movement distance	7 levels: 12.5%, 25%, 37.5%, 50%, 62.5%, 75%, 87.5% of screen width

**Figure 2.**
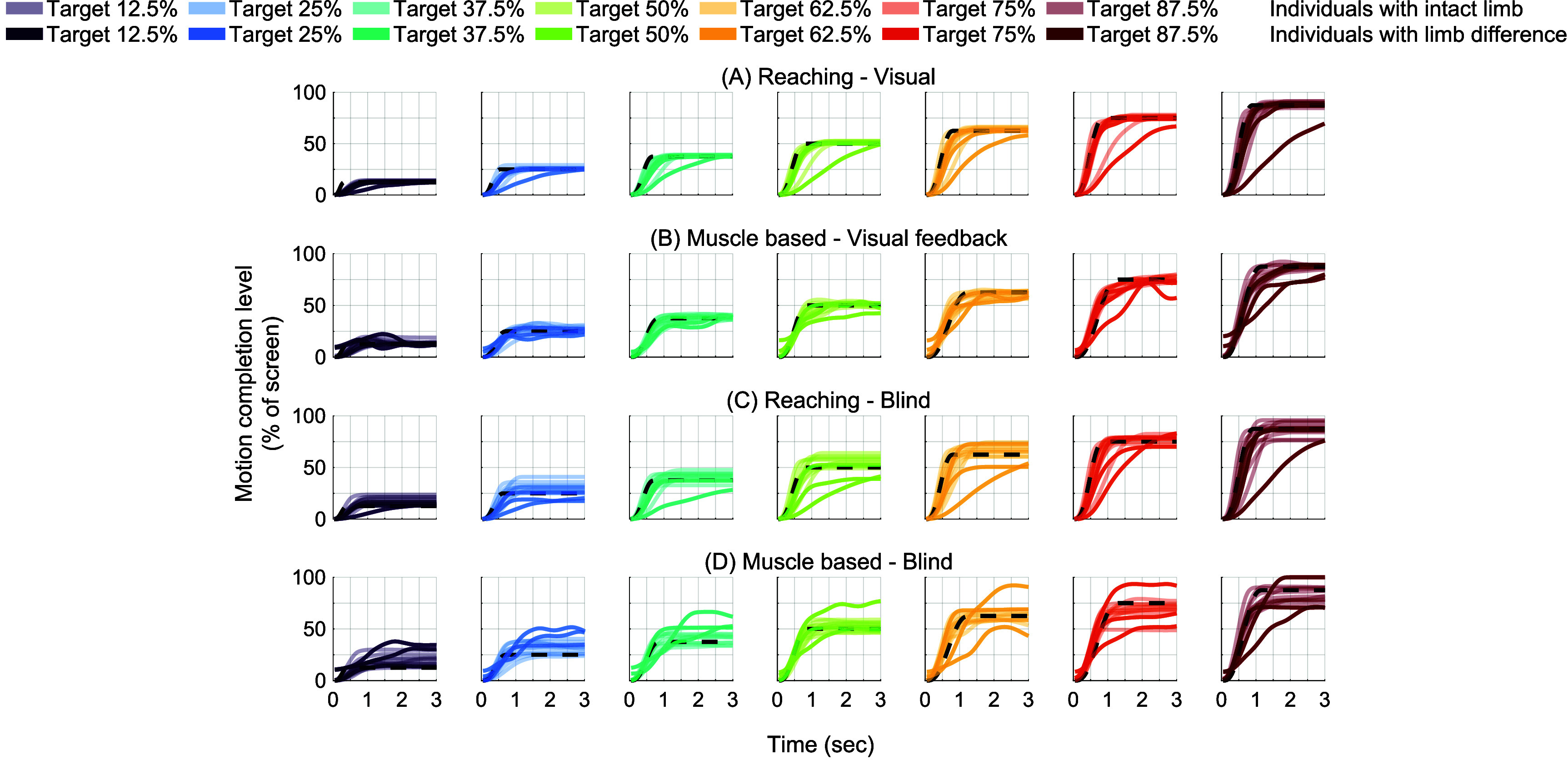
Position traces vs time. The dark colored lines represent position traces for the individuals with limb difference, while the lighter lines show the position traces for each individual with intact limb. The horizontal axis represents time (seconds) from movement onset, and the vertical axis represents the distance to the target as a percentage of the workspace. The dashed black line represents the minimum jerk trajectory.

For reaching conditions, the cursor location reflected the horizontal position of a manipulandum held by the participant. Participants made discrete reaching movements of varying amplitude with and without visual feedback. As shown in prior literature [10, 22, 23], these trajectories exhibited a single velocity peak near their midpoint and a bell-shaped velocity profile (figures 2(A)/(C), 3(A)/(C)).

For muscle control conditions, the cursor position was derived from the extent of muscle deformation as captured by sonomyographic imaging [13]. Participants generated cursor movement by performing wrist flexion or extension, again with and without visual feedback. These muscle-based trajectories also displayed a single velocity peak near the midpoint and a bell-shaped profile (figures 2(B)/(D), 3(B)/(D)).

### 3.1. Peak velocity

**Figure 3.**
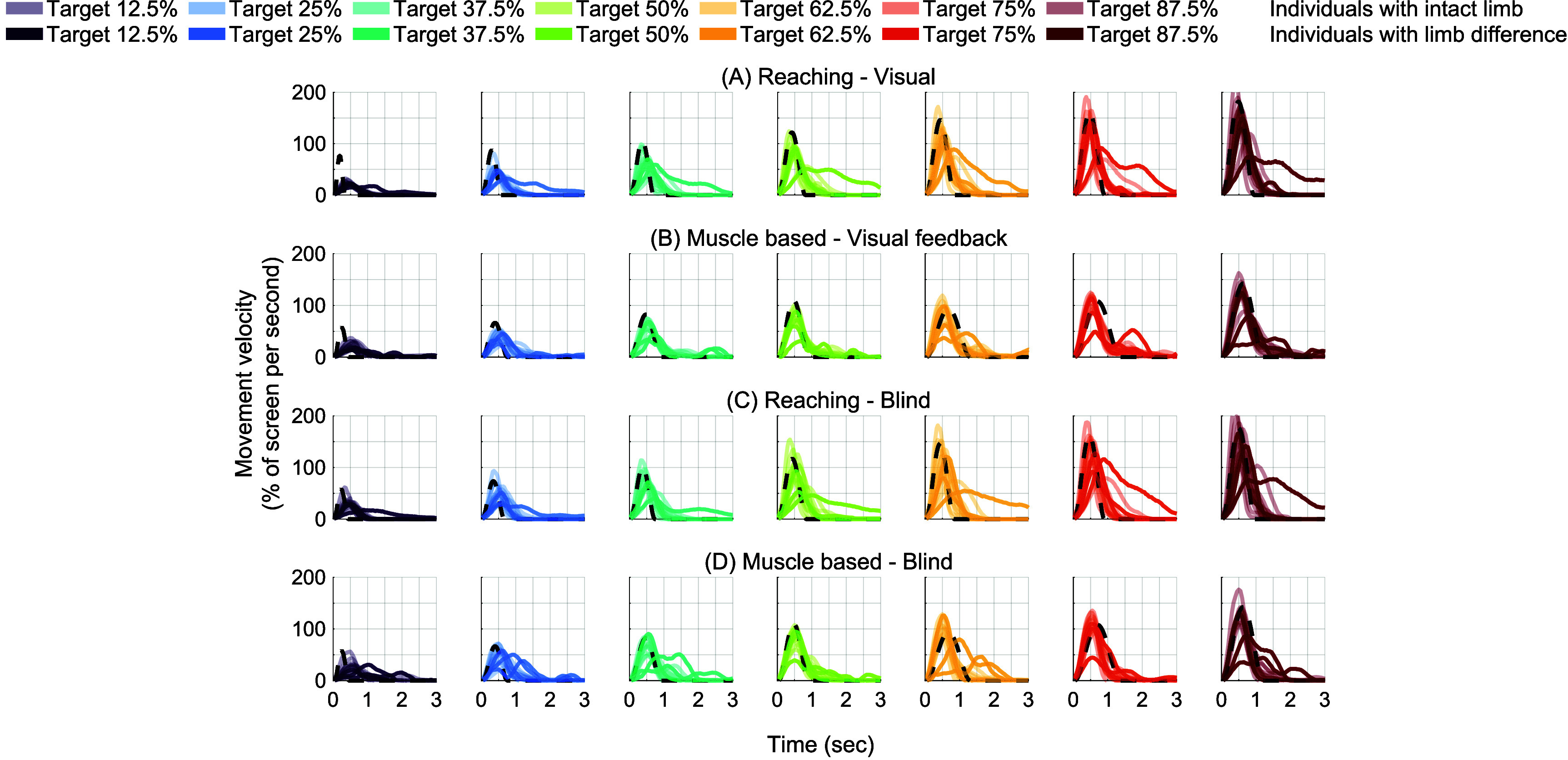
Velocity traces vs time. The dark colored lines represent velocity traces for the individuals with limb difference, while the lighter lines show the velocity traces for each individual with intact limb. The horizontal axis represents time (seconds) from movement onset, and the vertical axis represents the velocity of movement as a percentage of the workspace per second. The dashed black line represents MJT.

**Figure 4.**
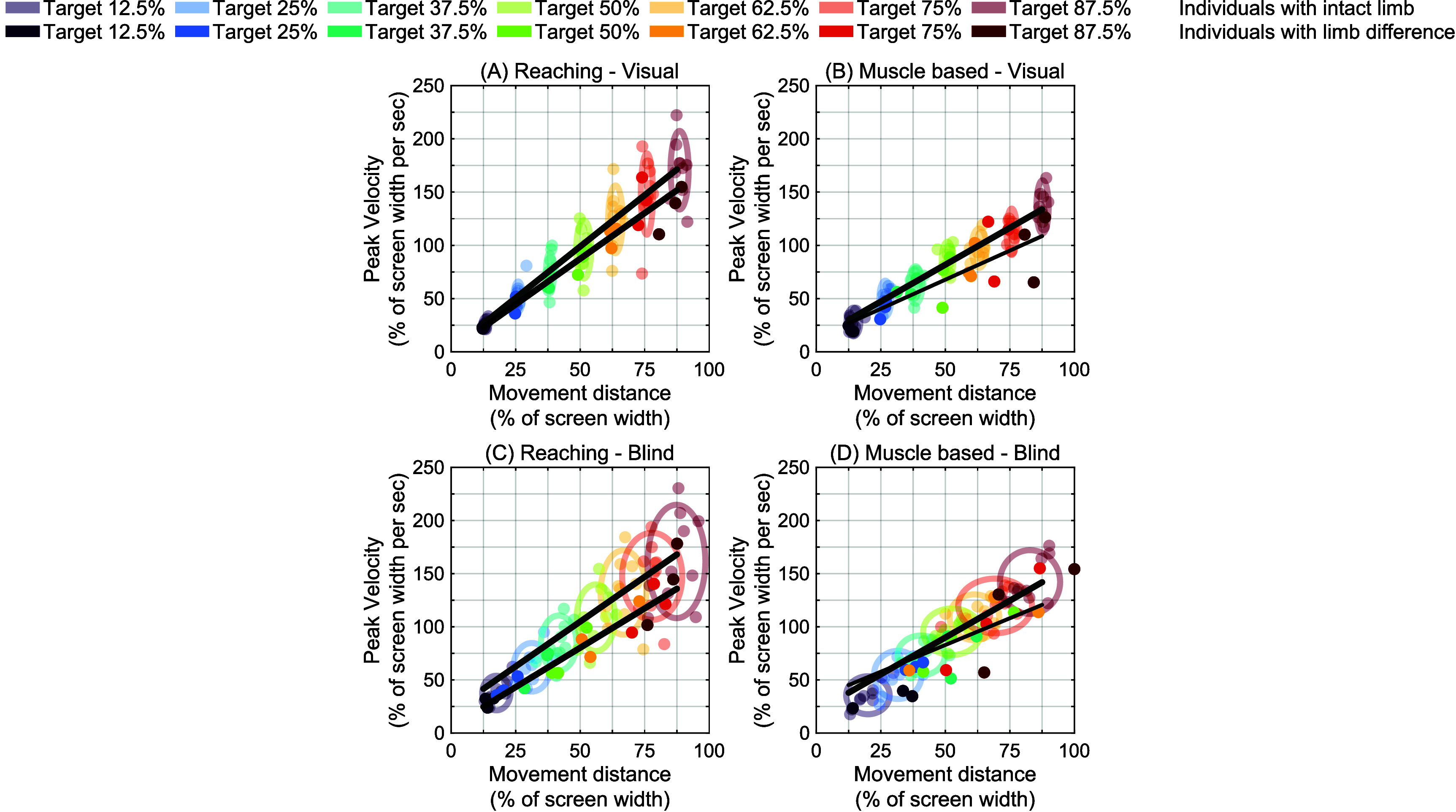
Peak velocity vs movement distance. The ellipse axes represent one standard deviation along each axis, the light colored points represent the underlying mean data for each participant, and the gray line represents the linear regression for individuals with intact limbs. The dark colored points represent data from individuals with limb difference, and the black line represents the corresponding linear regression. As the movement amplitude increased, the participants proportionally increased the peak movement velocity.

**Figure 5.**
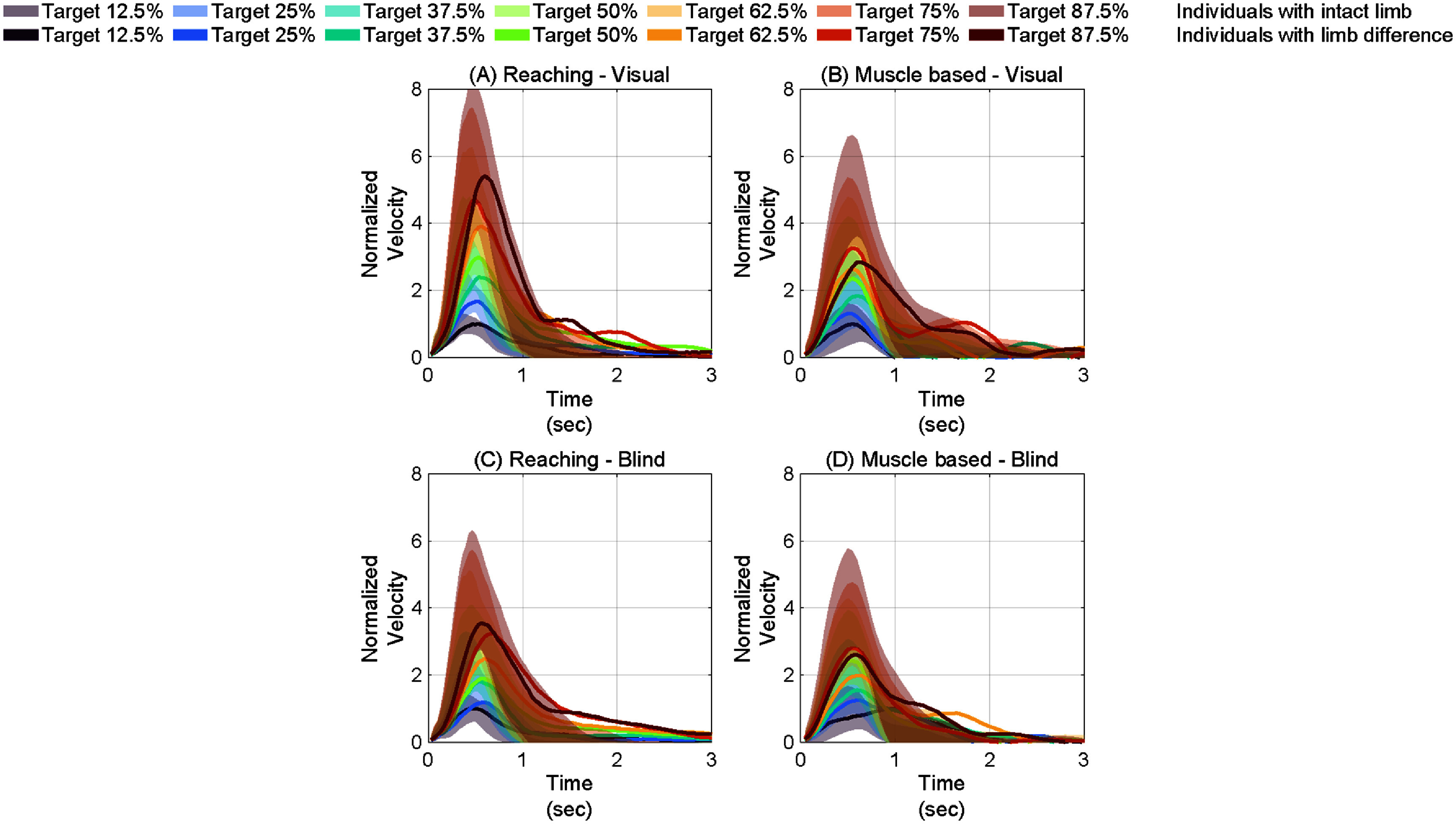
Mean velocity profiles across all participants vs time. The data is grouped by movement amplitude (each color represents a different movement distance). The shaded regions represent the individuals with an intact limb, while the lines represent the average of the three individuals with limb difference. Peak velocity increases with the movement distance.

SRH tests (table 3) revealed significant main effects of control modality ($H$ = 19.75, DF = 1, $p < $0.05), movement distance ($H$ = 1218.6, DF = 6, $p < $0.05), visual feedback condition ($H$ = 8.53, DF = 1, $p < $0.05), and limb status ($H =$ 33.82, DF = 1, $p < $0.05) on peak velocity. Peak velocities were higher for muscle-based control (Conditions C, D, G, H) than for reaching (Conditions A, B, E, F), and increased monotonically with target distance. Peak velocities were significantly higher when the visual feedback was absent and were also higher for individuals with intact limbs. No interaction terms reached significance. Linear regressions demonstrated consistent distance-dependent scaling of peak velocity across all conditions (figures 3–5). Slopes ranged from 1.27 to 1.98, with $R^2$ values between 0.7 and 0.9, indicating similar scaling for reaching and muscle-based control, regardless of visual feedback. Mean velocity traces (figures 3 and 5) revealed that both peak velocity and time to peak velocity increased systematically with target distance, consistent with the MJT model. The time to peak velocity for the farthest target increased 6.9 fold compared to the closest target during reaching and 6.6 fold during muscle control when visual feedback was available. Without visual feedback, it increased 4.98 fold for reaching and 6.1 fold for muscle control.

**Table 3.** Summary of statistical tests on performance measures.

Dependent variable	Factor tested	Test (DF, H/slope, $R^ {2}$)	$p$-value	Result/interpretation
Peak velocity	Control modality	SRH (1, $H =$ 19.75)	$ < $0.05	Higher for muscle-based
	Movement distance	SRH (6, $H =$ 1218.6)	$ < $0.05	Increased with distance
	Visual feedback	SRH (1, $H =$ 8.53)	$ < $0.05	Slightly higher for no visual feedback
	Intact limb presence	SRH (1, $H =$ 33.82)	$ < $0.05	Slightly higher for individuals with intact limb
	Interactions	SRH	$>$0.05	not significant
	Distance regressions	Linear (slopes 1.27–1.98, $R^2$ = 0.7–0.9)	$ < $0.05	Linear increase with movement distance across conditions

Time to target	Control modality	SRH (1, $H =$ 156.15)	$ < $0.05	Higher for reaching
	Movement distance	SRH (6, $H =$ 636.65)	$ < $0.05	Increased with distance
	Visual feedback	SRH (1, $H =$ 1.02)	$>$0.05	not significant
	Intact limb presence	SRH (1, $H =$ 66.53)	$ < $0.05	Slightly higher for individuals with limb difference
	Interactions	SRH	$>$0.05	not significant
	Distance regressions	Linear (slope = 0.01, $R^2$ = 0.5–0.8)	$ < $0.05	Linear increase with movement distance across conditions

Time to velocity peak	Control modality	SRH (1, $H =$ 239.05)	$ < $0.05	Higher for muscle-based
	Movement distance	SRH (6, $H =$ 17.56)	$ < $0.05	Increased with distance
	Visual feedback	SRH (1, $H =$ 5.29)	$<$0.05	Slightly higher for visual feedback absent
	Intact limb presence	SRH (1, $H =$ 114.56)	$ < $0.05	Higher for individuals with limb difference
	Interactions	SRH	$>$0.05	not significant

Position error	Control modality	SRH (1, $H =$ 10.153)	$ < $0.05	Higher for muscle-based
	Movement distance	SRH (6, $H =$ 63.83)	$ < $0.05	Increased with distance
	Visual feedback	SRH (1, $H =$ 47.71)	$ < $0.05	Higher for no visual feedback
	Intact limb presence	SRH (1, $H =$ 22.91)	$ < $0.05	Higher for individuals with limb difference
	Interactions	SRH	$>$0.05	not significant

### 3.2. Time to target

**Figure 6.**
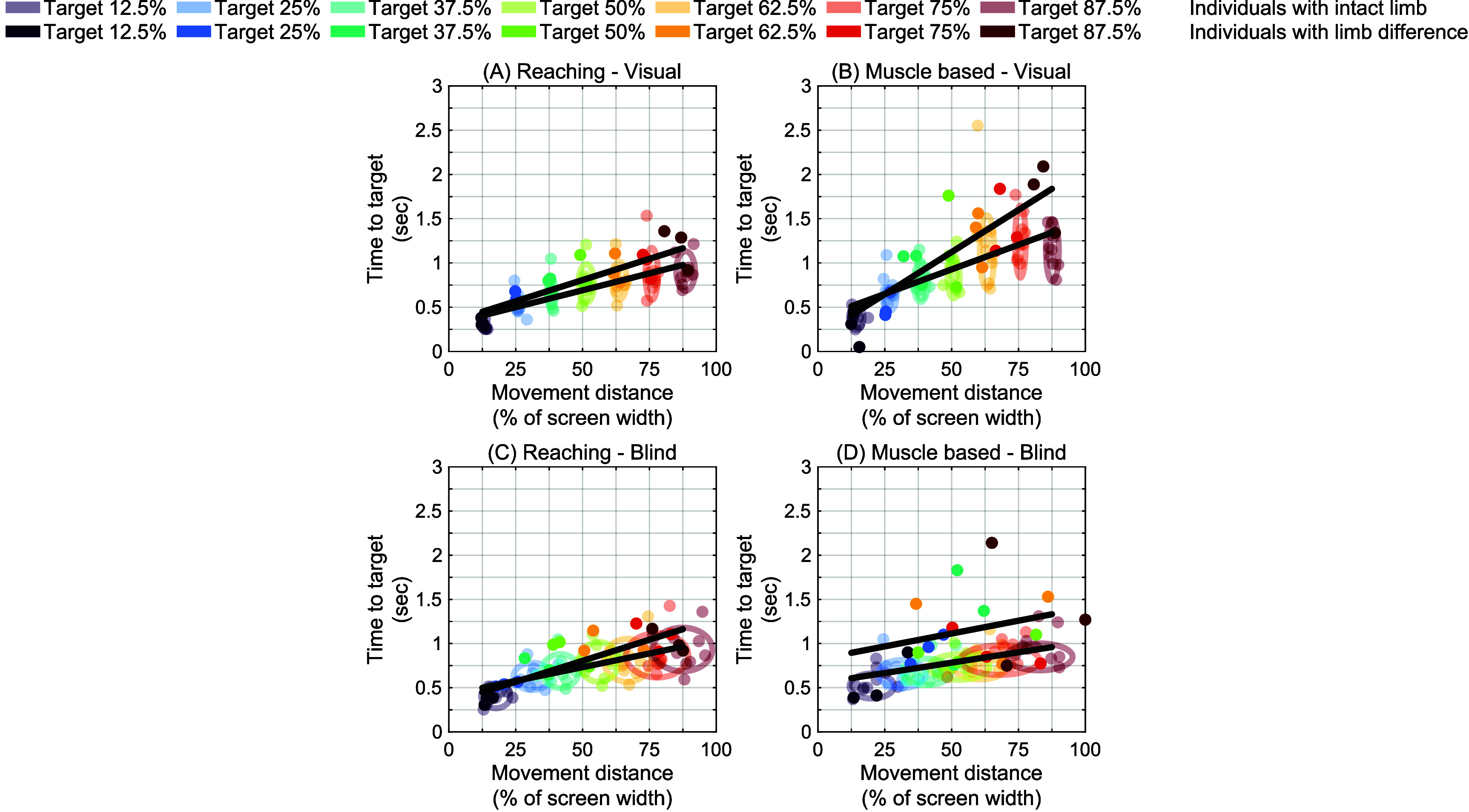
Time to target in seconds vs movement distance. The ellipse axes represent one standard deviation along each axis, the light colored points represent the underlying mean data for each participant, and the gray line represents the linear regression for individuals with intact limbs. The dark colored points represent data from individuals with limb difference, and the black line represents the corresponding linear regression. The time required to acquire a specific target increased as the movement distance increased.

SRH analysis showed significant effects of control modality ($H =$ 156.15, DF = 1, $p < $0.05), movement distance ($H =$ 636.65, DF = 6, $p < $0.05), and limb status ($H =$ 66.53, DF = 1, $p < $0.05), but not presence of visual feedback ($H =$ 1.02, DF = 1, $p < $0.05) on time to target. Muscle-based control yielded faster movements than reaching, and movements to farther targets required more time. Movements were slightly slower for individuals with limb difference. No interaction terms reached significance. Linear regressions demonstrated consistent distance scaling across all conditions (figure 6). Slopes were approximately 0.01 in all cases, with $R^2$ values ranging from 0.5 to 0.8.

### 3.3. Time to peak velocity

SRH tests revealed significant effects of control modality ($H =$ 239.05, DF = 1, $p < $0.05), movement distance ($H =$ 17.56, DF = 6, $p < $0.05), presence of visual feedback ($H =$ 5.29, DF = 1, $p < $0.05), and limb status ($H =$ 114.56, DF = 1, $p < $0.05) on the time taken to acquire velocity peak. Movements using muscle control reached peak velocity later, and longer distances required more time. It was higher when visual feedback was present and was also higher for individuals with limb difference. Interaction effects were not significant.

### 3.4. Position error

**Figure 7.**
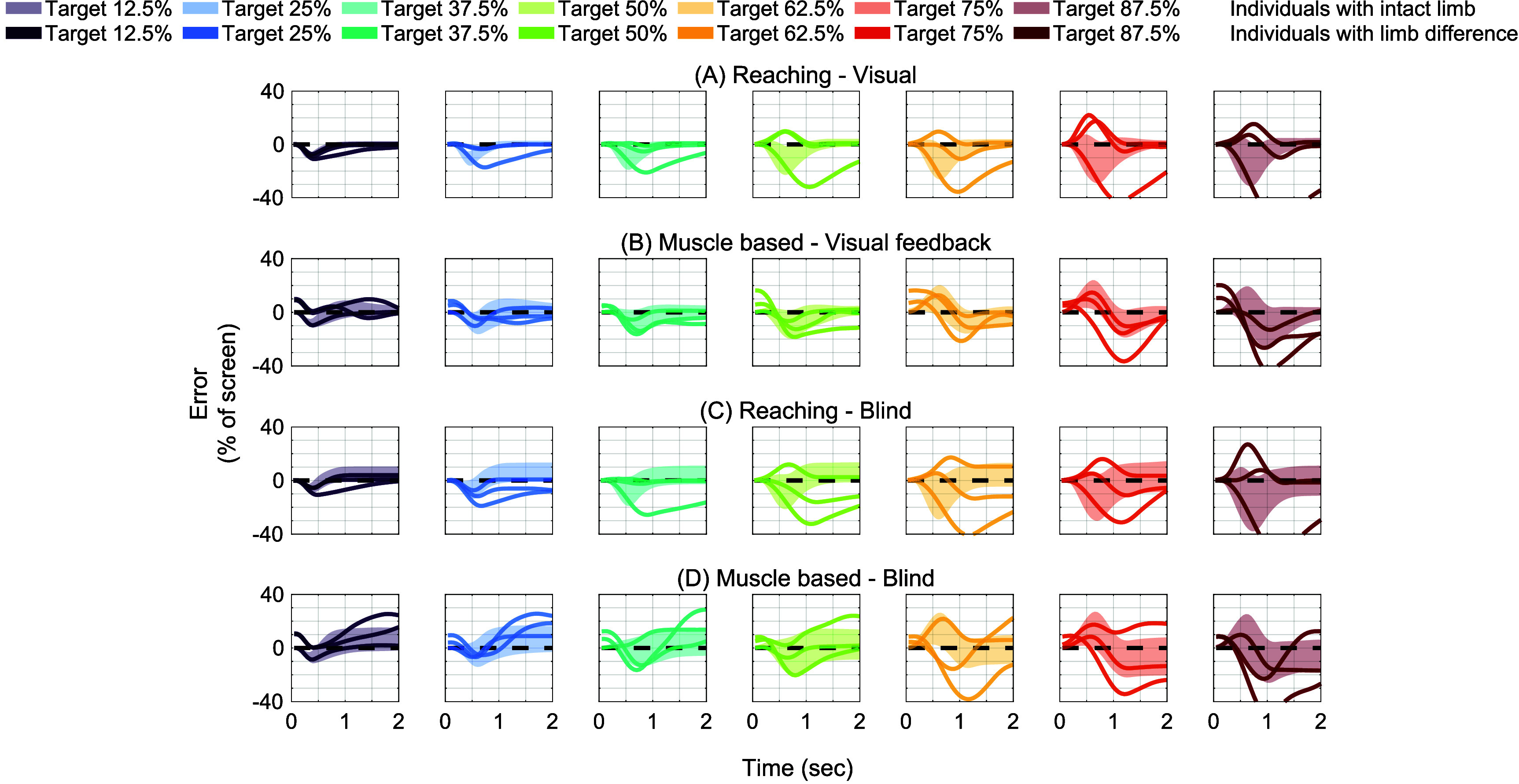
Error between cursor and ideal trajectory vs movement distance. The shaded region shows $\pm$ one standard deviation for the individuals with intact limb, while the lines represent the individuals with limb difference. The horizontal axis represents time (seconds) from movement onset, and the vertical axis represents percentage of the workspace.

**Figure 8.**
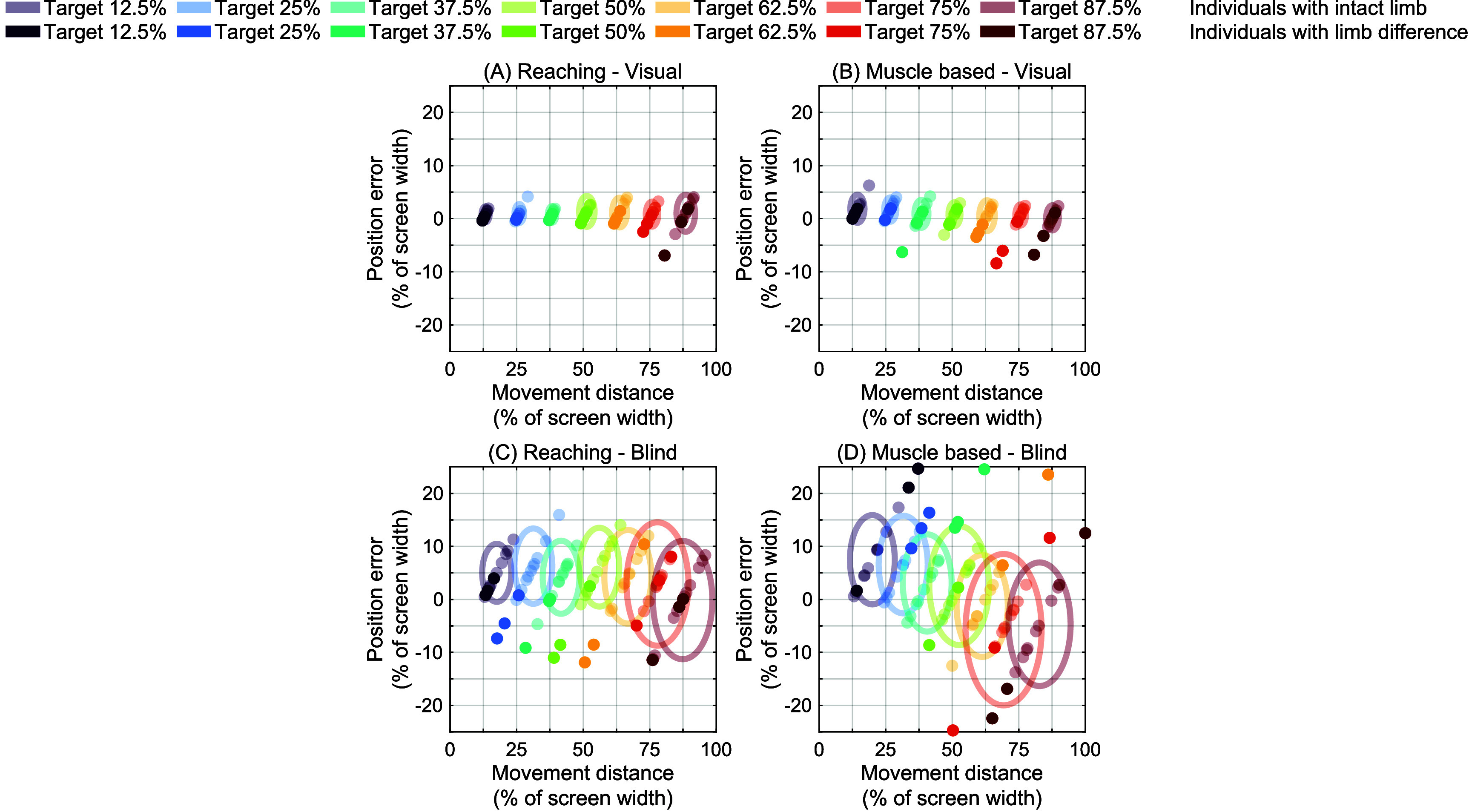
Position errors. Each point on the plot shows the position error for a trial plotted against movement distance for the corresponding trials. The ellipse axes represent one standard deviation along each axis and the light colored points represent the underlying mean data for each participant. The dark colored points represent data from individuals with limb difference. The position error was significantly lower when visual feedback was present across both control modalities.

SRH tests revealed significant effects of control modality ($H =$ 10.15, DF = 1, $p < $0.05), movement distance ($H =$ 63.83, DF = 6, $p < $0.05), presence of visual feedback ($H =$ 47.71, DF = 1, $p < $0.05), and presence of intact limb ($H =$ 22.91, DF = 1, $p < $0.05). Position error increased with movement distance and was greater for muscle-based control than reaching (figures 7 and 8). Absence of visual feedback increased position error, and it was slightly higher for individuals with limb difference. No interaction terms were significant.

### 3.5. Target completion rate

**Figure 9.**
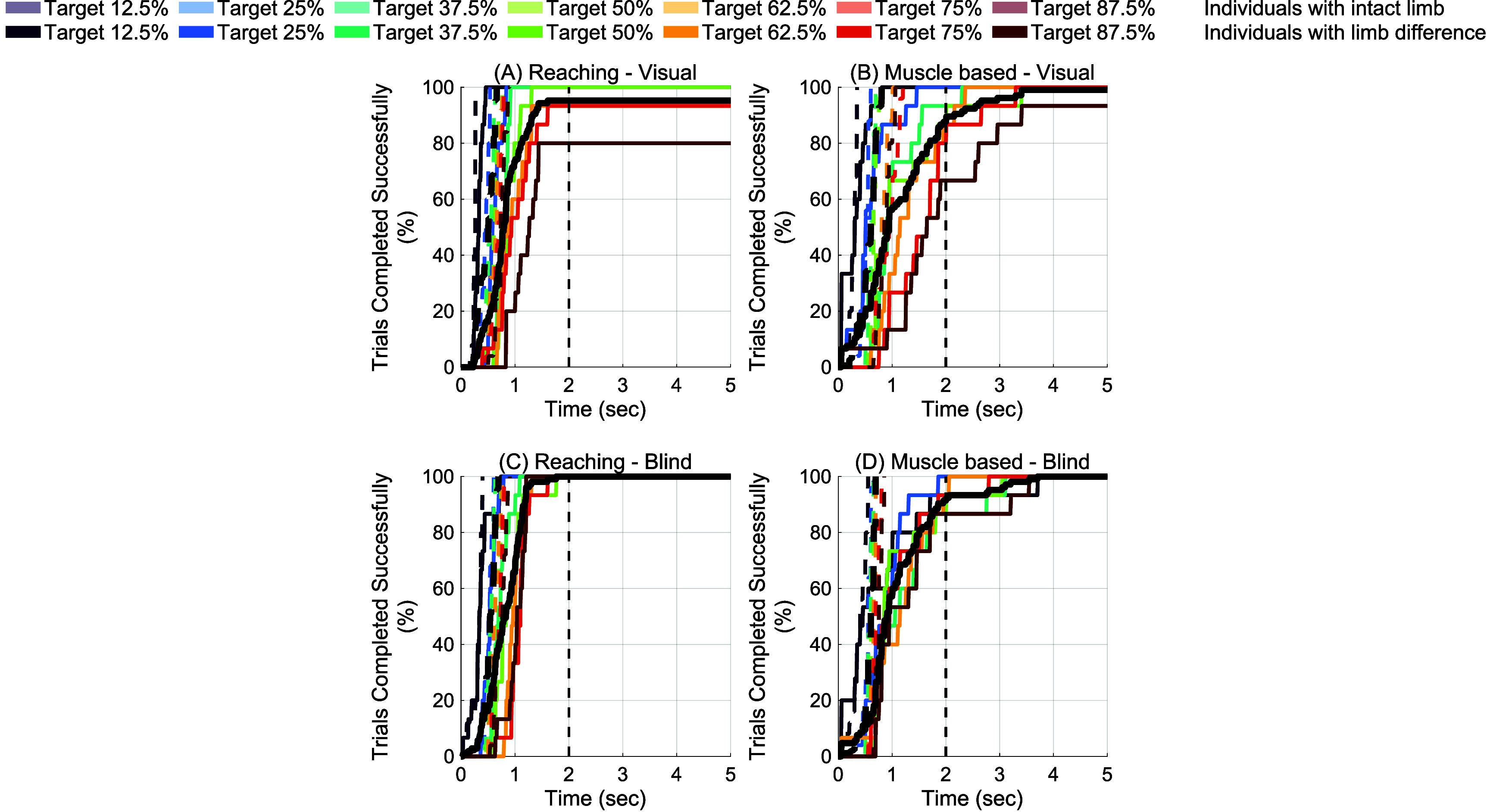
Percentage of completed trials as a function of time. Each color represents a movement distance, and the black line shows the average across all distances, which approaches completion within about two seconds. The main takeaway here is that participants completed almost all movements within roughly two seconds.

The percentage of targets successfully achieved within visual feedback trials remained high across both groups (figure 9). For reaching, individuals with intact limbs achieved 100% of targets, whereas individuals with limb differences achieved 95.2$\pm$8.2%. For muscle-based control, individuals with intact limbs achieved 100% of targets, and individuals with limb differences achieved 99.0$\pm$1.6%.

## 4. Discussion

The central contribution of this study is the demonstration that MJT-like profiles consistently emerge across diverse control modalities, the presence or absence of visual feedback, and across individuals with and without limb difference. These findings reveal that smooth, coordinated planning is preserved even when control signals are abstract, sensory feedback is limited, or natural limb dynamics are absent.

Importantly, the present study differs from prior work in several ways. First, we examined movement generation in individuals with limb difference, including both prosthesis mediated reaching and residual muscle based control. Second, we investigated trajectory formation under transformed control mappings in which cursor motion was not directly equivalent to hand motion in external space. Third, we examined MJT-like behavior during sonomyographic muscle based control, allowing trajectory formation to be studied at the level of muscle deformation rather than overt limb kinematics. Together, these findings suggest that smoothness based motor planning is robust across altered embodiment conditions, transformed sensorimotor mappings, and different control representations.

### 4.1. Minimum-Jerk trajectories emerge across control modalities

Our findings demonstrate that participants generated movement trajectories consistent with the MJT model across all experimental conditions. Regardless of whether the control modality involved traditional reaching or muscle-based control, participants exhibited smooth, bell-shaped velocity profiles and amplitude-dependent scaling of movement time and peak velocity. These results align with classical models of motor planning [1, 10] and extend prior work showing MJT adherence in control domains that are not directly tied to joint kinematics [11]. The consistency of these findings across modalities suggests that smoothness-based planning is preserved even when the control signal is extracted from abstract, muscle-driven control mappings, compared to directly from limb dynamics. These results were consistent across individuals with an intact limb as well as those with limb difference.

Although trajectories from individuals with intact limbs more closely followed the ideal MJT-like profiles, those from individuals with limb difference exhibited greater variability. This increased variability may arise from differences in sensorimotor mapping, prosthetic interface dynamics, and reduced fidelity of proprioceptive feedback. Importantly, the overall structure of the movement, including bell-shaped velocity profiles and distance-dependent scaling, remained consistent across groups.

Our findings should be interpreted in the context of prior work demonstrating that unconstrained reaching movements exhibit approximately straight hand trajectories and bell shaped velocity profiles consistent with minimum jerk predictions. Flash and Hogan [1, 10] further demonstrated that these trajectory properties persist even when direct visual information about the arm is reduced, suggesting that movement smoothness reflects generalized planning principles rather than continuous visual monitoring of limb position. The present findings are consistent with this broader literature and extend these observations to additional sensorimotor contexts.

### 4.2. Visual feedback is not necessary for trajectory smoothness

Typical point to point movement trajectories show a scaling of peak velocities and time to acquire targets based on target distance. A key result of this study is that visual feedback was not essential for preserving this scaling of peak velocity, as participants continued to produce MJT-like profiles even in the absence of visual feedback. Although visual feedback likely supports online refinement, its absence did not significantly disrupt the structure of the planned movement. This finding suggests that movement smoothness may emerge from internal models rather than external sensory corrections. While visual feedback did not significantly alter the overall smoothness of the trajectories, it did influence endpoint accuracy. Specifically, we observed larger endpoint errors in the blind conditions, consistent with the absence of visual correction. However, even in the presence of these systematic deviations from the intended target, the velocity profiles and trajectory shapes continued to closely follow the MJT model.

This raises an important insight: the MJT model appears to hold not because it necessarily maps the trajectory to the intended target position, but rather because it describes the smoothest transition between the starting point and the acquired final position. In earlier work, including our prior study [11] and foundational studies by Hogan and colleagues, MJT modeling was typically performed by substituting the final target position ($x_{f}$) into the analytical MJT equations (equations (2) and (3)). In conditions with visual feedback, the acquired position tends to closely match the target, and thus this substitution is valid and yields strong fits. However, in the absence of visual feedback, when endpoint errors become more pronounced, using the target position as the endpoint introduces a mismatch between the model and the observed data. When instead the actual acquired final position is used as $x_{f}$ in the MJT equations, the fit remains strong, even under blind conditions. This suggests that the MJT framework does not inherently depend on the correctness of the externally specified goal, but rather on the internal representation of the movement endpoint as realized by the participant.

In this sense, the MJT describes the executed motor output, and not necessarily the task-defined goal. We propose that this distinction is crucial when evaluating movement smoothness under conditions of sensory deprivation or altered control mappings. The central nervous system may optimize smoothness relative to the internally estimated endpoint, which may differ from the task target when feedback is limited. This interpretation reinforces the idea that MJT-like behavior is a robust feature of internally generated motor plans, even when accuracy is compromised. Furthermore, in the presence of visual feedback, the participant’s estimate of acquired position is more accurate, whereas in the absence of visual feedback, the end-point errors reflect the natural error between the internal model and measured kinematics.

It is important to note that, although visual feedback was removed in certain conditions, participants retained access to proprioceptive feedback arising from joint motion, muscle activation, and, in the case of individuals with limb difference, residual limb and socket interactions. Therefore, the experimental conditions do not isolate purely feedforward control, and we cannot directly dissociate the relative contributions of internal models and proprioceptive feedback. Accordingly, the persistence of MJT-like trajectories in the absence of visual feedback suggests that smooth movement structure is robust to the removal of visual information, rather than being independent of sensory feedback altogether. These results are consistent with the idea that internal models operate in conjunction with proprioceptive feedback to support movement planning and execution.

### 4.3. Individuals with limb difference maintain smooth control

Notably, individuals with upper-limb difference produced trajectories that were qualitatively and quantitatively similar to those of participants with intact limbs. These individuals were able to perform both reaching and muscle-based control tasks with comparable trajectory profiles, despite lacking feedback from the missing limb. This suggests that internal movement representations can persist and support smooth planning even when natural afferent pathways are disrupted. Although the results are quite consistent across the individuals with limb difference and those with intact limbs, we recognize that the number of individuals with limb difference recruited for this study is a limitation of this work.

### 4.4. Muscle-based control enables generalizable planning

Our results also support the hypothesis that MJT-like trajectories are preserved even when the motor system operates in an abstract control domain. Sonomyographic control relies on voluntary modulation of muscle contraction and is decoupled from traditional joint movement, yet participants still produced coordinated and smooth trajectories. This indicates that the characteristic features of MJT-like behavior can be maintained across different levels of control, ranging from joint-level limb movements to muscle-level contraction signals, and across the corresponding differences in motor output. From a motor learning perspective, this suggests that smoothness reflects a deeply rooted organizing principle of the motor system that generalizes across interfaces and output domains.

In the muscle-based control condition, position errors exhibited a directional bias that was not observed in reaching movements. This asymmetry may be due to the mapping between muscle deformation and cursor position, which may not be perfectly linear or symmetric across directions. Factors such as differences in activation between flexor and extensor muscles, as well as calibration of the control signal, may introduce systematic biases in cursor movement. As a result, participants may consistently over- or undershoot targets in one direction, leading to the observed directional error patterns.

The emergence of bi-directional error at larger movement distances during muscle-based control may also reflect limitations in the control signal used for muscle-based control. In the current implementation, cursor position was derived from muscle deformation signals that primarily captured activity from a subset of forearm muscles, rather than explicitly measuring both agonist and antagonist muscle groups independently. Because precise movement termination typically relies on coordinated activation of antagonistic muscles, this reduced representation may limit the ability to finely regulate endpoint position, particularly for larger movements. This could contribute to the observed pattern of both overshooting and undershooting at increased movement distances.

### 4.5. Implications for assistive interfaces and prosthetics

These findings have important implications for the design of assistive technologies. The ability to generate naturalistic and smooth trajectories even in the absence of joint movement or sensory feedback suggests that muscle-driven interfaces can support intuitive control for individuals with motor impairments or limb difference. Muscle-based systems may therefore serve as a promising control option in situations where mechanical motion is restricted or sensory feedback is unreliable. Such situations include operating a robotic end effector for remote manipulation, controlling a biomechatronic device during critical tasks, or actuating prosthetic limbs and other assistive devices. By demonstrating that smooth motor control strategies are preserved under these conditions, this work helps establish a foundation for prosthetic and assistive interfaces that draw directly on internal motor representations.

### 4.6. Implications for studying loss of motor control

Hogan [1] proposed that the central nervous system selects the MJT during the path-planning stage when a target is pre-selected. Subsequent work has shown that similar behavior occurs across a wide variety of tasks, including arm reaching, catching, drawing, vertical arm movements, head movements, saccadic eye movements, chewing, and other motor skills [4–10, 24–29]. Highly coordinated multi-joint upper limb movements have also been shown to exhibit minimum-jerk trajectories [3, 9, 10, 30]. However, this relationship for smooth movements breaks down in neurologically impaired individuals, such as those with stroke. These patients often exhibit reduced movement smoothness, deviations from a MJT, and delayed corrective movements to achieve certain end-points [31–33]. Consequently, rehabilitation robots have been designed to train users to perform minimum-jerk movements following stroke [34]. Our current approach opens a completely new window to study these motor control principles at the muscle level. Using this technique, it is now possible to investigate how different neurological conditions manifest at the level of muscle activity.

Motor control at the level of individual muscles provides insight into how coordinated movements are organized. It has been shown that complex movements can be decomposed into structurally elemental, independent [35] sub-movements, often referred to as muscle synergies [36, 37]. These synergies have been observed across multiple levels of motor control [38–41] and reflect dynamic spatiotemporal patterns of activation among groups of muscles working together to produce coordinated movement. Typically, electromyography is used to study the firing patterns of individual muscles or groups of muscles [35, 42–44], and various decomposition or source localization methods [45–47] are applied to identify independent contributions that combine to generate complex motions. Muscle deformation signals as used in this study can open a new window into studying motor control at the muscle level. Additionally, recent work has shown that multimodal time-synchronized analysis of sEMG and sonomyography signals can also be of value, especially while studying deficits at multiple levels of the neuromuscular signal chain [48].

Muscle synergies are altered in certain populations with neurological impairments [49–51], demonstrating the importance of muscle-level organization for smooth and efficient movement. By examining these patterns, we can better understand how individual muscles and small muscle groups contribute to coordinated motor behavior, providing a framework for studying both normal and impaired motor control.

### 4.7. Limitations

Several limitations should be noted. First, the sample size for individuals with limb difference was small, which may limit generalizability. Second, the task was constrained to one degree-of-freedom in a cursor-control environment. Although this allowed for precise analysis of trajectories, real-world motor tasks often involve multiple degrees-of-freedom and more complex coordination demands. Third, the study did not include perturbations or adaptation phases that might reveal how feedback contributes to error correction or motor learning over time. Lastly, while sonomyographic control was effective in this task, sensor placement and stability could affect robustness in uncontrolled settings. We plan to expand our participant pool to include more individuals with limb difference, increase the degrees-of-freedom in the task to examine whether MJT-like behaviors generalize to more complex movements, integrate additional or multimodal sensing modalities to further enhance the robustness and applicability of our control interfaces, and determine how learning, adaptation, and fatigue influence performance. Incorporating neuroimaging or electrophysiological measures could help elucidate how internal models are represented and maintained across different sensory and control conditions.

An additional limitation is the heterogeneity of prosthesis experience within the limb difference group. Participants differed substantially in prosthesis use history, including years of experience and type of prosthetic control system used. Such differences may influence internal representations of prosthetic dynamics, familiarity with socket interactions, and motor adaptation strategies. As a result, movement trajectories may differ between experienced and naive prosthesis users. Future studies with larger participant cohorts will be needed to systematically investigate how prosthesis experience and control modality influence MJT-like trajectory formation and motor planning behavior.

An important consideration in interpreting the present results is the relationship between the sensing modality and the control mapping used to generate cursor movement. In the current study, sonomyography was selected because it enables proportional position based control through direct measurement of muscle deformation. In contrast, EMG systems [52–55] commonly implement velocity based control schemes in which activation amplitude modulates movement speed rather than cursor position directly. Differences in control strategy and signal characteristics may therefore influence movement trajectories and endpoint regulation. Although the present findings demonstrate that MJT-like trajectories emerge during sonomyographic muscle based control, it remains unclear to what extent these trajectory properties generalize across other biosignal modalities such as EMG. It is possible that smoothness based trajectory structure depends not only on the underlying neural planning process, but also on how motor intent is transformed into control output. Future studies directly comparing position based and velocity based control mappings across different sensing modalities may help clarify how interface design influences movement smoothness and trajectory formation.

In summary, this study shows that smooth, MJT-like movement trajectories emerge robustly across variations in control modality, sensory feedback availability, and limb status. These results support the hypothesis that internal motor planning strategies drive the execution of movement, independent of external feedback or control paradigm. By extending the MJT framework to muscle-based control in individuals with and without limb difference, this work provides insight for the development of intuitive, biologically relevant control systems.

## 5. Conclusion

Decades of work on limb kinematics has shown that human movements follow highly stereotyped, smooth trajectories. In our previous work, we demonstrated that these canonical patterns are preserved even at the level of isolated muscle-based control. The present findings further show that such trajectories persist without visual feedback, across different control modalities, and even in individuals with limb absence, suggesting that movement smoothness reflects a fundamental property of internal motor planning rather than peripheral implementation.

## Data Availability

The data cannot be made publicly available upon publication because no suitable repository exists for hosting data in this field of study. The data that support the findings of this study are available upon reasonable request from the authors.
